# Apoptosis-Inducing Effects of Short-Chain Fatty Acids-Rich Fermented Pistachio Milk in Human Colon Carcinoma Cells

**DOI:** 10.3390/foods12010189

**Published:** 2023-01-01

**Authors:** Su-Jin Lim, Hyuk-Cheol Kwon, Dong-Min Shin, Yong-Jun Choi, Seo-Gu Han, Yea-Ji Kim, Sung-Gu Han

**Affiliations:** 1 Department of Food Science and Biotechnology of Animal Resources, Konkuk University, Seoul 05029, Republic of Korea; 2 Research Group of Food Processing, Korea Food Research Institute, Wanju 55365, Republic of Korea; 3 Department of Animal Science and Technology, Konkuk University, Seoul 05029, Republic of Korea

**Keywords:** pistachio milk, pistachio, plant-based milk, short-chain fatty acids, apoptosis, microtubule, colon carcinoma, anti-cancer activity

## Abstract

Pistachio milk (PM), an extraction product of pistachio, is protein- and fat-dense food. Short-chain fatty acids (SCFAs) are known for inducing cytotoxicity and apoptosis in colon carcinoma cells. This study aimed to find an optimal combination of probiotics that can produce a higher amount of SCFAs in PM. In addition, the anti-cancer effect of fermented PM on human colon carcinoma cells (Caco-2) was determined. The combinations of probiotics were as follows: *Streptococcus thermophilus* + *Lactobacillus bulgaricus* (C); C + *Lactobacillus acidophilus* (C-La); C + *Lactobacillus gasseri* (C-Lg); C + *Bifidobacterium bifidum* (C-Bb). The results indicated that fermented PM was produced after a short fermentation time in all the probiotics combinations. C-Bb produced up to 1.5-fold more acetate than the other probiotics combinations did. A significant amount of cytotoxicity, i.e., 78, 56, and 29% cell viability was observed in Caco-2 cells by C-Bb-fermented PM at 1, 2.5 and 5%, respectively. C-Bb-fermented PM (5%) induced early and late apoptosis up to 6-fold. Additionally, Caco-2 cells treated with C-Bb-fermented PM significantly induced the downregulation of α-tubulin and the upregulation of cleaved caspase-3, as well as nuclear condensation and fragmentation. Our data suggest that fermented PM, which is rich in acetate, may have the potential as a functional food possessing anti-colon cancer properties.

## 1. Introduction

Plant-based milks, which are non-dairy beverages derived from plant sources, are increasingly consumed around the world as substitutes for bovine milk [1]. Animal products cause a negative impact on the environment such as greenhouse-gas emission, and bovine milk in particular has health concerns for those with lactose intolerance and a milk protein allergy, leading to its declining demand [2,3]. Plant-based milks are safe to be consumed by those with a milk intolerance or allergy due to the lack of lactose and allergens, such as casein [1,2]. Additionally, the increasing number of vegetarians is also associated with the increase in the consumption of plant-based milks [2,4,5]. Plant-based milks are mainly produced from nuts and oilseeds (e.g., almond, cashew nut, coconut, peanut, soybean, and walnut), which are preferred by vegetarians [1].

Plant-based milks possess many antioxidants and fatty acids that can lead to positive health effects, including a decrease in the risk of cardiovascular diseases, cancer, atherosclerosis, and diabetes [6]. However, the nutritional quality of most plant-based milk is lower than that of bovine milk [7]. Therefore, the selection of raw materials containing high protein and fat contents is important in the development of plant-based milk [8]. 

Pistachio (*Pistacia vera* L.) is a popular and nutritious tree nut. It is globally consumed due to its high nutritional value, health properties, and sensory characteristics [9]. Particularly, pistachios have a high level of unsaturated fatty acids and a low level of saturated fatty acids, and they are a rich source of protein, dietary fibers, vitamins, and minerals [10]. Thus, pistachios are an appropriate plant source for the production of plant-based milk. In fact, pistachios are utilized to make food products such as pistachio milk (PM), pistachio butter, pistachio paste, and pistachio spreads [11,12,13,14]. Importantly, pistachios contain many functional compounds including high levels of lutein, γ-tocopherol, and phenolic compounds, suggesting that they have antioxidant and anti-inflammatory effects [15]. However, previous studies have focused only on the production optimization and quality improvement of PM, rather than its functional properties [11,12].

The fermentation of plant-based milks can enhance their functional properties by increasing the level of bioactive compounds [16]. For example, the fermentation of brown rice milk with *Lactobacillus pentosus* increased the amount of gamma-aminobutyric acid in it [17]. Soy milk fermented with *Lactobacillus paracasei* showed high antioxidant activity from aglycones that were generated from isoflavone glucosides [18]. The fermentation of pistachios was known to increase the short-chain fatty acids (SCFAs) level [19]. It was reported that pistachios fermented with human fecal microbiota have higher SCFAs than fermented hazelnuts, almonds, macadamia, and walnuts do. Meanwhile, fermented plant-based milks (e.g., soy milk, rice milk, and coconut milk) can be suitable carriers of probiotic strains [20]. 

*Lactobacillus*, *Bifidobacterium*, and *Streptococcus* are mainly utilized in the fermentation of plant-based milk [20]. *Streptococcus thermophilus* and *Lactobacillus delbrueckii ssp. bulgaricus* were used as commercial yogurt starters for both cow milk and soy milk [21]. *Lactobacillus* species (e.g., *L. acidophilus*, *L. plantarum*, and *L. rhamnosus*) and *Bifidobacterium* species (e.g., *B. longum*, *B. breve*, and *B. lactis*) are major probiotics in fermented foods [22]. This is because these probiotics can produce beneficial substances including linoleic acid, vitamin, lactic acid, bacteriocin, and bioactive peptides. In addition, *Lactobacillus* and *Bifidobacterium* strains can metabolite pyruvate to SCFAs, mainly acetate, propionate, and butyrate during fermentation [23]. For example, dairy yogurt fermented with *Lactobacillus acidophilus*, *Lactobacillus gasseri*, or *Bifidobacterium bifidum* showed a higher level of SCFA content [24]. SCFAs formed by bacterial fermentation have numerous intestinal health effects including the inhibition of inflammatory activity, the decrease in pathogenic microorganisms, the stimulation of mucin production, and cancer cell apoptosis [25]. 

Colon cancer is the third most common cancer worldwide. Most colon cancers are sporadic and occur as a result of a somatic genetic mutation due to exposure to dietary factors such as excessive ingestion of red and processed meat and a deficient intake of dietary fiber, whole grains, and other healthy nutrients [26,27]. In other words, diet may prevent and treat most colon cancers [27]. Fermented foods have various components (e.g., bioactive polysaccharides and peptides, SCFAs, and phenolic compounds) that may reduce the risk of cancer [28]. For example, fermented soybean extract containing bioactive isoflavones reduced the colony formation and growth of colon carcinoma cells and increased apoptotic cell death [29]. Additionally, recent studies have demonstrated that the increased production of SCFAs during fermentation exerted anti-cancer properties against colon carcinoma cells [30,31,32]. 

SCFAs, especially butyrate, are known to induce apoptosis in cancer cells through the inhibition of histone deacetylase (HDAC) [33]. According to past studies, the suppression of HDAC induced histone hyperacetylation, and in turn, caused chromatin remodeling and affected DNA replication, repair, and transcription [33,34]. Acetate does not possess a strong HDAC inhibition potency, although it is produced in higher concentrations than other SCFAs (i.e., butyrate and propionate) are [33,35]. On the other hand, SCFAs can affect microtubules composed of α- and β-tubulin heterodimers [34]. A previous study determined that SCFAs can change the microtubule formation and function in colon carcinoma cells through increasing the amount of tubulin expression [36]. Microtubule change, especially α-tubulin, is associated with cytotoxicity and apoptotic cell death in colon carcinoma cells [37,38,39]. 

Therefore, this study aimed to evaluate the fermentation property of PM, particularly SCFA production. The PM was fermented with probiotics that are known to produce SCFAs. In addition, the functional properties and underlying mechanisms of fermented PM were determined in human colon carcinoma cells (Caco-2) for apoptotic cell death.

## 2. Materials and Methods

### 2.1. Materials and Chemicals

The pistachios and bovine milk were purchased from Theone b&f Co., Ltd. (Gyeonggi, Korea) and Seoul Dairy Cooperative (Seoul, Korea), respectively. The inulin was purchased from Vixxol Co., Ltd. (Gyeonggi, Korea). The lactobacilli MRS broth and agar powder were provided by Becton, Dickinson and Company (Sparks, MD, USA) and Duksan Co., Ltd. (Gyeonggi, Korea), respectively. The acetate and lactate were supplied by Daejung Chemicals & Metals Co., Ltd. (Gyeonggi, Korea) and Kanto Chemical Co., Ltc. (Tokyo, Japan), respectively. The L-cysteine, propionic acid, and n-butyric acid were supplied by Junsei Chemical Co., Ltd. (Tokyo, Japan). The pivalic acid was obtained from Tokyo Chemical Industry (TCI) Co., Ltd. (Tokyo, Japan). Dulbecco’s modified Eagle’s medium (DMEM), fetal bovine serum (FBS), penicillin/streptomycin, and 0.05% trypsin/0.53 mM ethylenediaminetetraacetic acid (EDTA) solution were supplied by WELGENE Inc. (Gyeongsan, Korea). The phosphate-buffered saline (PBS) was provided by Lonza (Walkersville, MD, USA). The T-75 flasks, 6-well plates, and 12-well plates were obtained from SPL Life Sciences (Pocheon, Gyeonggi, Korea). The nitrocellulose membrane was purchased from GE Healthcare Bio-Sciences (Pittsburgh, PA, USA). The 4,6-diamidino-2-phenylindole dihydrochloride (DAPI) was supplied by Sigma-Aldrich (St. Louis, MO, USA). The antibodies against alpha-tubulin and Casepase-3 were purchased from Cell Signaling Technology (Beverly, MA, USA) and Santa Cruz Biotechnology Inc. (Santa Cruz, CA, USA), respectively. The antibody against glyceraldehyde 3-phosphate dehydrogenase (GAPDH) was obtained from EMD Millipore (Burlington, MA, USA).

### 2.2. Lactic Acid Bacteria Strains

*Streptococcus salivarius* ssp. *thermophilus* KCTC 3779, *Lactobacillus delbrueckii* subsp. *bulgaricus* KCTC 3635, *Lactobacillus acidophilus* KCTC 3171, *Lactobacillus gasseri* KCTC 3163, and *Bifidobacterium bifidum* KCTC 3202 were supplied by the Korean Collection for Type Cultures (Jeongeup, Korea). *Streptococcus thermophilus*, *Lactobacillus bulgaricus*, *Lactobacillus acidophilus*, and *Lactobacillus gasseri* were kept on MRS agar and streaked every two weeks. Prior to their experimental use, these four strains were subcultured twice in an MRS broth at 37 °C for 16 h. *Bifidobacterium bifidum* was kept on MRS agar supplemented with 0.05% cysteine in anaerobic conditions and streaked every two weeks. Prior to its experimental use, *B. bifidum* was subcultured twice in MRS broth supplemented with 0.05% cysteine in anaerobic condition at 37 °C for 48 h.

### 2.3. Preparation of Pistachio Milk

**Figure 1:**
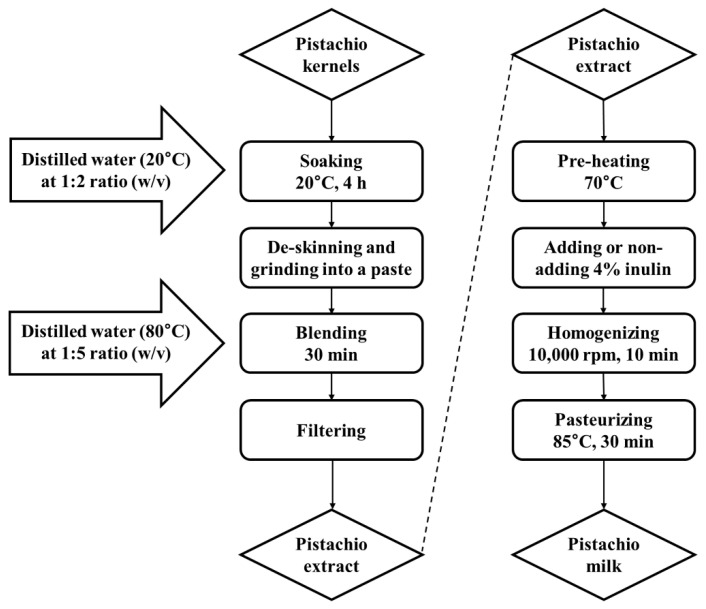
The process for the production of pistachio milk (PM).

The PM was prepared as previously described with few modifications [11]. The process of producing PM was illustrated in Figure 1. The pistachio kernels were first soaked in distilled water at a 1:2 ratio (*w*/*v*) at 20 °C for 4 h. Next, the soaked pistachio kernels were de-skinned and ground into a paste using a bowl cutter (C4 VV, Sirman, Venezia, Italy). The pistachio paste was blended with hot distilled water (80 °C) at a 1:5 ratio (*w*/*v*) for 30 min. A pistachio extract was prepared after the mixture had been filtered with a muslin cloth. The pistachio extract was preheated at 70 °C, and then homogenized at 10,000 rpm for 10 min with or without 4% inulin using a Model AM-7 homogenizer (Nissei Co., Lid., Tokyo, Japan). Then, the homogenized pistachio extract was pasteurized at 85 °C for 30 min. The PM was stored at 4 °C for the subsequent experiments.

### 2.4. Proximate Composition Analysis

The pistachio kernels were ground into powder using a mortar. The dry matter and ash content of the pistachios, PM, and bovine milk were determined following previous study methods with minor modifications [40,41]. Briefly, the samples were weighed in an aluminum weigh dish and ceramic crucible, respectively. Next, the samples in the aluminum weigh dish were dried in an oven (SW-09D, Sangwoo scientific Co., Bucheon, Korea) to measure the dry matter content. To measure the ash content, the samples in the ceramic crucible were burned in a muffle furnace. Prior to the ashing procedure, the PM and bovine milk were placed in a dry oven at 100 °C for 1 h. Then, the samples in the aluminum weigh dish and ceramic crucible were cooled to 25 °C and weighed again. The conditions for dry matter and ash content measurements are shown in Table 1. The protein, fat, and dietary fiber contents of the pistachios and PM were determined following the Association of Official Analytical Chemists’ (AOAC) methods 950.48 (1995), 922.06 (1995), and 991.43 (1990), respectively [42,43]. In addition, the sugar content of the pistachios and PM were calculated by subtracting the percentage of the total protein, fat, ash, and dietary fiber contents from the percentage of dry matter content. Meanwhile, the protein, fat, and sugar (lactose) content of the bovine milk were measured using MilkoScan Minor (Foss, Hilleroed, Denmark). 

### 2.5. Fermentation of Pistachio Milk 

The fermented PM was prepared as previously described with minor modifications [24]. The PM was preheated at 42 °C in a water bath, and then, it was inoculated with probiotics. The combinations of probiotics were as follows: (1) *S. thermophilus* and *L. bulgaricus*, used as a conventional yogurt starter combination (C; 1.5 × 10^7^ cfu/mL of each probiotics); (2) *S. thermophilus*, *L. bulgaricus*, and *L. acidophilus* (C-La; 1 × 10^7^ cfu/mL of each probiotics); (3) *S. thermophilus*, *L. bulgaricus*, and *L. gasseri* (C-Lg; 1 × 10^7^ cfu/mL of each probiotics); (4) *S. thermophilus*, *L. bulgaricus*, and *B. bifidum* (C-Bb; 1 × 10^7^ cfu/mL of each probiotics). *L. acidophilus*, *L. gasseri*, and *B. bifidum* were employed because these are known to produce high amounts of SCFAs [23,44]. The inoculated PM was incubated at 42 °C until the pH reached 4.6, and then, it was stored at 4 °C overnight for further experiments.

### 2.6. Preparation of Supernatants for Pistachio Milk and Fermented Pistachio Milk

The supernatants of the PM and fermented PM were obtained for cell culture experiments and gas chromatography (GC), as described previously [24]. The supernatants were separated by centrifuging the PM and fermented PM samples (10 g) twice at 3000× *g* for 20 min at 4 °C and then at 10,000× *g* for 30 min at 4 °C. For the cell culture experiment, the supernatants of the PM and fermented PM were filtered using a 0.45-μm syringe filter (Advantec, Tokyo, Japan). For the GC analysis, 1 mL of filtered supernatants of PM and fermented PM was added with 200 µL of 25% meta-phosphoric acid, and then, they were centrifuged at 20,000× *g* for 20 min. The supernatants containing meta-phosphoric acid were filtered through a 0.45-μm syringe filter.

### 2.7. Measurements of Acidification Kinetics Parameters

The pH the fermented PM was measured using a pH meter (F-71, HORIBA, Kyoto, Japan) during fermentation at 20 min intervals. The acidification kinetic parameters were determined by a method used in a previous study [45]. The maximum acidification rate (V_max_) was calculated from the change in pH over time (dpH/dt), and it is expressed as 10^−3^ pH units/min. At the end of the fermentation, the time required to reach V_max_ (t_max_), the time taken to reach pH 5.0 (t_pH5.0_), and the time taken to complete fermentation (t_f_) were calculated.

### 2.8. Gas Chromatography (GC) Analysis

The SCFAs and lactate contents in the PM and fermented PM supernatants were determined using GC (HP 6890, Hewlett-Packard Co., CA, USA) using a flame ionization detector (FID). The used column was a fused silica capillary column (30 m × 0.32 mm × 0.25 µm; Cat No. 2-4131, SUPELCO, PA, USA). The supernatants of the PM and fermented PM (1 mL) were mixed to 50 µL of 2% pivalic acid, and then, they were transferred to a vial, respectively. Pivalic acid was used as an internal standard. A standard solution was produced by adding acetate, propionate, butyrate, lactate, and pivalic acid into deionized water. One microliter of the supernatant sample was injected into the inlet of gas chromatography (split 1:70). The oven temperature program was as follows: the oven temperature initially set at 100 °C for 1 min, it was increased by 10 °C/min to 150 °C, it was held at 150 °C for 7 min, it was increased by 20 °C/min to 180 °C, and then it was held at 180 °C for 1 min. The flow rate of the carrier gas (helium) was maintained at 1.5 mL/min. 

### 2.9. Cell Culture and Treatments

The human colon carcinoma cell line (Caco-2 cells) was obtained from the Korean Cell Line Bank (Seoul, Korea). The cells were cultured in DMEM supplemented with 10% FBS and 1% penicillin/streptomycin (*v*/*v*) at 37 °C in a humidified carbon dioxide (CO_2_) incubator containing 5% CO_2_. The culture medium was changed every 2 days, and the cells were subcultured every 3–4 days at a density of 21 × 10^5^ cells/flask (75 cm^2^) using a 0.05% trypsin/0.53 mM EDTA solution. The cells were seeded in 6-well plates and 12-well plates at a density of 1.5 × 10^5^ cells/well and 1.0 × 10^5^ cells/well, respectively. Then, the plates were incubated for 24 h before being treated with fermented PM supernatants, acetate, and lactate for 48 h. The Caco-2 cells between passages 36 and 50 were used in the experiments.

### 2.10. Cytotoxicity Assay 

Cytotoxicity was determined by a trypan blue dye exclusion test. The Caco-2 cells were seeded in 6-well plate and treated with the fermented PM supernatants (0.5, 1, 2.5, and 5%), acetate (0, 5, 10, 20, and 30 mM), or lactate (0, 5, 10, 20, 30, and 40 mM) for 48 h. In addition, deionized water (5%) was used totreat the Caco-2 cells as a control. The cells were detached using a 0.05% trypsin/0.53 mM EDTA solution and centrifuged at 187× *g* for 5 min [46]. The pellets were resuspended in PBS and dyed with trypan blue. The number of viable cells was counted manually using a hemocytometer. 

### 2.11. Cell Morphologic Observation

The Caco-2 cells were seeded in 6-well plate and treated with the fermented PM supernatants (0.5, 1, 2.5, and 5%), acetate (0, 5, 10, 20, and 30 mM), or lactate (0, 5, 10, 20, 30, and 40 mM) for 48 h. In addition, deionized water (5%) was used as a control. After washing them twice with PBS, the cells’ morphology was captured using a Nikon Eclipse Ts2R camera (Nikon Co. Ltd., Tokyo, Japan).

### 2.12. Annexin V/Propidium Iodide Assay for Determination of Apoptosis

Cell apoptosis was determined using the EzWayAnnexin V-FITC Apoptosis Detection Kit (Sigma-Aldrich, St. Louis, MO, USA) as described previously with minor modifications [47]. The Caco-2 cells were seeded in 6-well plate and treated with fermented PM supernatants (1, 2.5, and 5%), acetate (0, 10, 20, and 30 mM), or lactate (0, 20, 30, and 40 mM) for 48 h. In addition, deionized water (5%) was used as a control. Next, the cell medium was collected in a 15 mL tube, and the cells were washed twice using PBS. Then, the cells were harvested with a 0.05% trypsin/0.53 mM EDTA solution and centrifuged at 2000× *g* for 5 min at 4 °C. After removing the medium supernatants, the cells were washed twice with cold serum-containing media and PBS, and then, they were resuspended in 1× binding buffer. Subsequently, the cells were added with 1.25 µL of Annexin V-fluorescein isothiocyanate and 1 µL of propidium iodide (1 mg/mL), and then, they were incubated in the dark for 30 min at 4 °C. Next, the cells were centrifuged at 1000× *g* for 5 min at 4 °C and re-suspended again in 1× binding buffer. The re-suspended cells were analyzed immediately using a CytoFlex Flow Cytometry Analyzer (Beckman Coulter, Brea, CA, USA).

### 2.13. Western Blot Analysis for Determination of Protein Expression Level of Microtubule- and Apoptosis-Related Markers

The expression levels of the microtubule- and apoptosis-related proteins were determined by a Western blot analysis [48]. The Caco-2 cells were seeded in 6-well plate and treated with the fermented PM supernatants (1, 2.5, and 5%), acetate (0, 5, 10, 20, and 30 mM), or lactate (0, 5, 10, 20, 30, and 40 mM) for 48 h. Deionized water (5%) was used as a control. The cells were collected and lysed using a RIPA-buffer and a protease inhibitor cocktail (Abbkine, Wuhan, China) on ice. The cell lysates were centrifuged at 23,500× *g* for 20 min at 4 °C, and the protein concentrations of the supernatants were measured using a Pierce BCA protein assay kit (Sigma-Aldrich, MO, USA). The Western blot protein samples (15 µg) were separated by sodium dodecyl sulfate-polyacrylamide gel electrophoresis (SDS-PAGE) and transferred to 0.2-μm pore-size nitrocellulose membranes. The membranes were blocked with 3% non-fat milk buffer for 1 h 30 min, and then, they were incubated with primary antibodies overnight at 4 °C. The membranes were washed and then incubated with horseradish peroxidase-conjugated secondary antibody for 1 h 30 min. The protein bands were visualized using an enhanced chemiluminescence (ECL) detection reagent (Thermo Fisher Scientific, MA, USA). The density of the protein bands was estimated using ImageJ software (National Institute of Health, Bethesda, MD, USA). In addition, the membranes were reused after the stripping method, as previously described [45]. The membranes were incubated with a stripping buffer at 57 °C for 30 min and then washed thrice with distilled water for 5 min. After being washed again with Tris-buffered saline with Tween 20 for 10 min, the membranes were blocked with 3% non-fat milk buffer for Western blot analysis.

### 2.14. Immunofluorescence Microscopy for Evaluation of Microtubules and Apoptosis

The alternations of the microtubules and nuclei were evaluated by immunofluorescence staining [47]. The Caco-2 cells were seeded in 12-well plate and treated with the fermented PM supernatants (1, 2.5, and 5%) and acetate (30 mM) for 48 h. Deionized water (5%) was used as a control. The cells were fixed with 4% paraformaldehyde for 15 min and washed thrice with 0.1% tween 20 in PBS. Next, the cells were permeabilized using 0.1% Triton X-100 in PBS for 10 min and washed thrice with 0.1% tween 20 in PBS. Subsequently, the cell monolayers were incubated with a blocking buffer composed of 3% bovine serum albumin and 2% normal donkey serum for 1 h and then with anti-alpha-tubulin diluted in the blocking buffer for 16 h at 4 °C. After washing them thrice with 0.1% tween 20 in PBS, the cells were incubated with DyLightTM-488-conjugated-IgE for 1 h 30 min at 25 °C. Then, the cells were washed thrice with 0.1% tween 20 in PBS, which was followed by their fixation with 4% paraformaldehyde for 10 min. After the cell nuclei were stained with DAPI (1 μg/mL) for 10 min, the cells were washed thrice with 0.1% tween 20 in PBS. Images of fluorescence labeled cells were captured using a Nikon Eclipse Ts2R camera.

### 2.15. Statistical Analysis

All the data are expressed as mean ± standard error. The statistical significance was evaluated by an independent 2-sample student’s t-test and a one-way analysis of variance (ANOVA) with Tukey’s post hoc test and Dunnett’s test, respectively. The tests were performed using SPSS-PASW statistics software version 18.0 (SPSS Inc., IL, USA). The statistical differences were considered significant at *p*-value < 0.05.

## 3. Results

### 3.1. Proximate Composition of Pistachio, Pistachio Milk, and Bovine Milk

The proximate compositions of pistachio, PM, and bovine milk are shown in Table 2. The PM had approximately ten times lower dry matter and protein contents, a seven times lower fat content, and a five times lower ash content than the pistachio did. However, the PM had a similar protein content and higher fat content when it was compared with that of the bovine milk. With regard to the sugars and dietary fiber content, the PM had considerably lower total carbohydrates than the pistachio and bovine milk did. For the fermentation of the PM, 4% inulin was added to the PM to supplement the level of sugars in the bovine milk.

### 3.2. Changes of pH and Acidification Kinetics of Fermented Pistachio Milks

**Figure 2:**
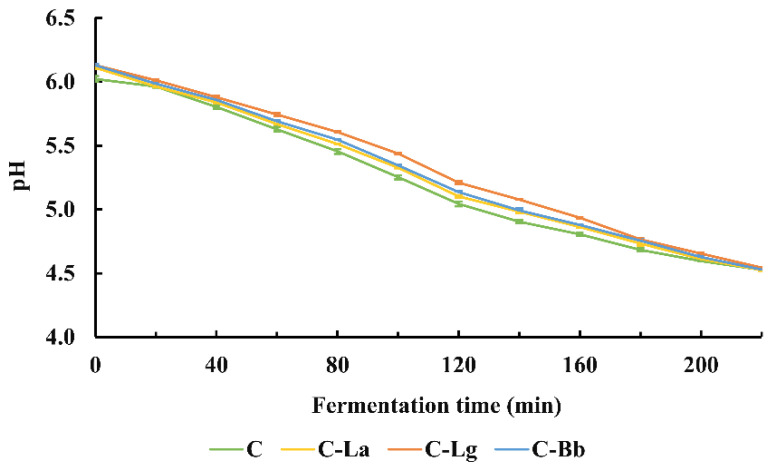
Changes in pH of fermented PMs during fermentation time. C = PM fermented with *Streptococcus thermophilus* and *Lactobacillus bulgaricus*; C-La = PM fermented with *S. thermophilus*, *L. bulgaricus*, and *Lactobacillus acidophilus*; C-Lg = PM fermented with *S. thermophilus*, *L. bulgaricus*, and *Lactobacillus gasseri*; C-Bb = PM fermented with *S. thermophilus*, *L. bulgaricus*, and *Bifidobacterium bifidum*. Results are expressed as mean ± standard error (*n* = 3).

The pH of the C-La-, C-Lg-, and C-Bb-fermented PM groups was slightly higher than that of C-fermented PM group during fermentation (Figure 2). In addition, the C-La, C-Lg, and C-Bb groups had a slightly lower V_max_ value and higher t_max_, t_ph 5.0_, and t_f_ values than the C group did (Table 3). The C-La, C-Lg, and C-Bb groups showed similar values for V_max_ and t_f_. However, the C-La and C-Bb groups had lower t_max_ and t_ph 5.0_ values than the C-Lg group did.

### 3.3. Concentrations of SCFAs and Lactate in Pistachio Milk and Fermented Pistachio Milk

**Figure 3:**
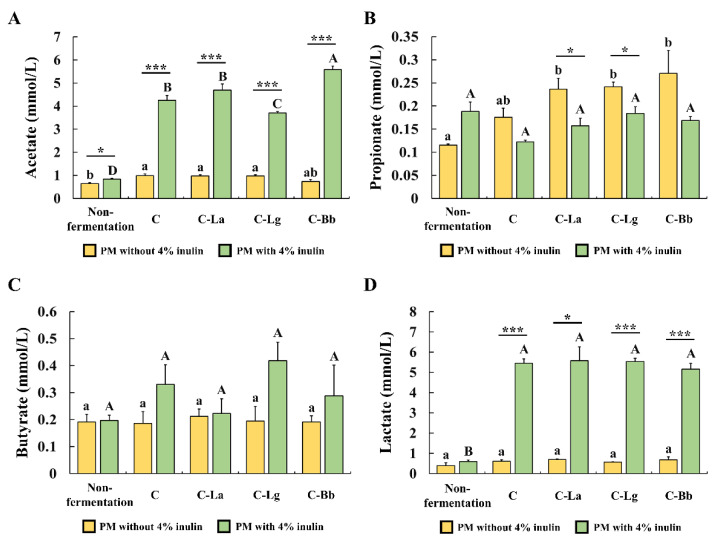
The concentrations of SCFAs ((**A**) acetate, (**B**) propionate, and (**C**) butyrate), and (**D**) lactate in PMs and fermented PMs with or without 4% inulin as measured by gas chromatography. C = PM fermented with *Streptococcus thermophilus* and *Lactobacillus bulgaricus*; C-La = PM fermented with *S. thermophilus*, *L. bulgaricus*, and *Lactobacillus acidophilus*; C-Lg = PM fermented with *S. thermophilus*, *L. bulgaricus*, and *Lactobacillus gasseri*; C-Bb = PM fermented with *S. thermophilus*, *L. bulgaricus*, and *Bifidobacterium bifidum*. Results are expressed as mean ± standard error (*n* = 3). Means with different letters (a–b) represent significant differences among PM without inulin (*p* < 0.05). Means with different letters (A–D) indicate significant differences among PM with 4% inulin (*p* < 0.05). * (*p* < 0.05) and *** (*p* < 0.001) indicate significant differences in two PM groups with and without 4% inulin.

The concentrations of SCFAs (acetate, propionate, and butyrate) and lactate in the PM and fermented PM with or without 4% inulin were analyzed using GC (Figure 3). The GC chromatograms are shown in Supplementary Figure S1. The fermented PM groups containing 4% inulin had higher acetate, butyrate, and lactate contents than the fermented PM without 4% inulin did. The propionate content was not greatly influenced by the addition of inulin, fermentation, and the combination of probiotics. The concentration of acetate (ranging from 3.70 to 5.58 mmol/L) and lactate (ranging from 5.15 to 5.58 mmol/L) was significantly affected by the addition of 4% inulin in all of the fermentation groups (Figure 3A,D). The butyrate levels were also increased by fermentation with 4% inulin in some of the probiotics combinations (Figure 3C). The C-Bb group fermented with 4% inulin had the highest acetate content compared to the other groups (Figure 3A). There was no significant difference in the lactate concentrations among the fermented PM groups with 4% inulin (Figure 3D). Therefore, the C-Bb group (fermentation of the PM added with 4% inulin and a combination of probiotics (*S. thermophilus*, *L. bulgaricus*, and *B. bifidum*)) was used for further experiments.

### 3.4. Cytotoxicity and Morphological Changes in Human Colon Carcinoma Cells (Caco-2 cells) Treated with Fermented Pistachio Milk, Acetate, and Lactate

**Figure 4:**
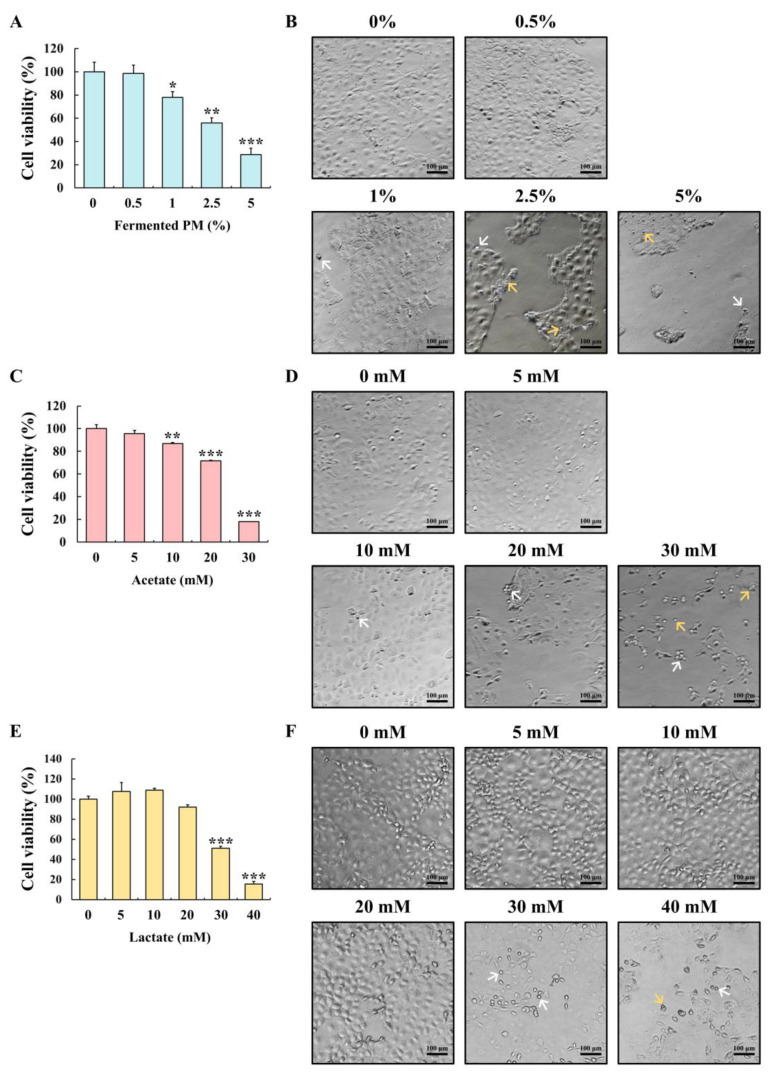
Effects of fermented PM, acetate, and lactate on cell viability and morphology. The cell viability treated with (**A**) fermented PM, (**C**) acetate, and (**E**) lactate in Caco-2 cells. The cell morphology treated with (**B**) fermented PM, (**D**) acetate, and (**F**) lactate in Caco-2 cells. The magnification of the cell morphology image was 100 ×. Scale bar = 100 µm. White arrows indicate rounded cells. Yellow arrows indicate rounded and shrunk cells. The cells were treated with fermented PM (0, 0.5, 1, 2.5, and 5%), acetate (0, 5, 10, 20, and 30 mM), and lactate (0, 5, 10, 20, 30, and 40 mM) for 48 h (*n* = 3 wells per groups). Results are expressed as mean ± standard error (*n* = 3). * (*p* < 0.05), ** (*p* < 0.01), and *** (*p* < 0.001) indicate significant differences compared with the control (0% and 0 mM).

The treatment of the Caco-2 cells with fermented PM, acetate, and lactate significantly decreased the viable cell numbers compared to the control in a concentration-dependent manner (*p* < 0.05; Figure 4A,C,E). At a lower concentration of these treatments (i.e., fermented PM at 0.5%, acetate at 5 mM, and lactate at 5, 10, and 20 mM), the viability of the Caco-2 cells was not significantly affected. The half-maximal inhibitory concentrations (IC_50_) of fermented the PM, acetate, and lactate on the Caco-2 cells were 2.5%, 22 mM, and 30 mM, respectively. In the cell morphology observations, the fermented PM (1, 2.5, and 5%), acetate (10, 20, and 30 mM), and lactate (30 and 40 mM) groups showed damaged cells (Figure 4B,D,F). In the fermented PM-treated cells, the round-shaped cells appeared at 1% fermented PM, and the rounded and shrunk cells were found at over 2.5% fermented PM. The empty space increased due to detached and floated cells in the wells. The rounded, shrunk, detached, and floated forms of the cells were also found in the cells treated with acetate (10, 20, and 30 mM) and lactate (30 and 40 mM).

### 3.5. Determination of Apoptosis in Caco-2 Cells Treated with Fermented Pistachio Milk, Acetate, and Lactate

**Figure 5:**
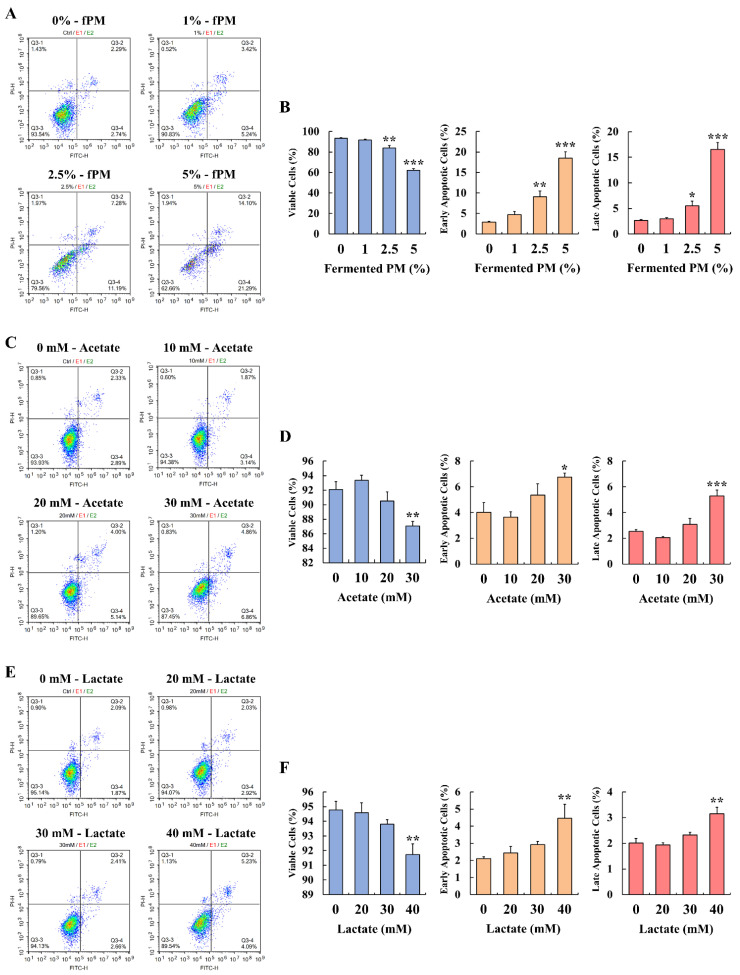
Effects of fermented PM (fPM), acetate, and lactate on cell viability and apoptosis in Caco-2 cells. Flow cytometry scatter plots of propidium iodide (y-axis) versus annexin V-fluorescein isothiocyanate (x-axis) on treatment with (**A**) fermented PM, (**C**) acetate, and (**E**) lactate in Caco-2 cells. Images of flow cytometry scatter plots are representative of three independent experiments. Percentage of viable cells, early apoptotic cells, and late apoptotic cells treated with (**B**) fermented PM, (**D**) acetate, and (**F**) lactate in Caco-2 cells. The cells were treated with fermented PM (0, 1, 2.5, and 5%), acetate (0, 10, 20, and 30 mM), and lactate (0, 20, 30, and 40 mM) for 48 h (*n* = 3 wells per groups). Results are expressed as mean ± standard error (*n* = 3). * (*p* < 0.05), ** (*p* < 0.01), and *** (*p* < 0.001) indicate significant differences compared with the control (0% and 0 mM).

In the treatment of the Caco-2 cells with the fermented PM, acetate, and lactate, the apoptotic ratios increased compared to that of the control (Figure 5A,C,E). At a higher concentration of these treatments (i.e., fermented PM at concentrations of 2.5% and 5%, acetate at a concentration of 30 mM, and lactate at a concentration of 40 mM), the Caco-2 cells were significantly affected (*p* < 0.05; Figure 5B,D,F). The cells treated with fermented PM (2.5 and 5%) had significantly lower viable cells and significantly higher early and late apoptotic cells compared to those of the control (Figure 5A,B). Specifically, after the treatment of the cells with fermented PM (2.5 and 5%), the number of early apoptotic cells was remarkably increased to 9.05 ± 1.44% and 18.48 ± 1.56%, respectively. Moreover, the fermented PM (5%) increased the number of late apoptosis cells by up to 16.51 ± 1.37%. Acetate and lactate also induced apoptotic cell death (Figure 5C–F). Acetate (30 mM) and lactate (40 mM) significantly decreased the viable cells and increased the early and late apoptotic cells compared to that which occurred in the control. In the treatment of the cells with acetate at a 30 mM concentration, the number of early and late apoptotic cells was increased to 6.75 ± 0.33% and 5.30 ± 0.46%, respectively. At 40 mM of lactate, the number of early and late apoptotic cells was increased to 4.46 ± 0.82% and 3.15 ± 0.25%, respectively.

### 3.6. Evaluation of α-tubulin Protein Expression in Caco-2 Cells Treated with Fermented Pistachio Milk, Acetate, and Lactate

**Figure 6:**
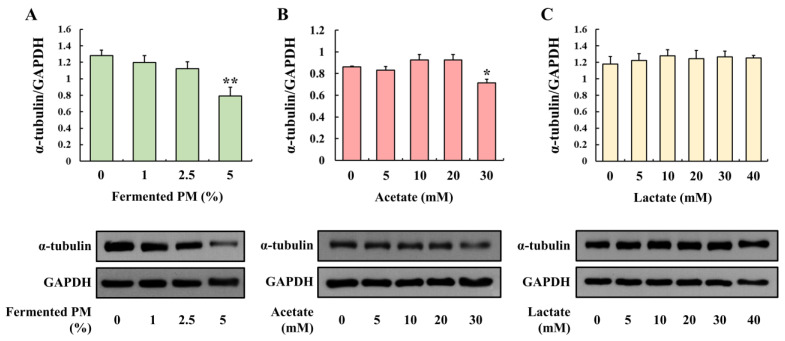
Effect of fermented PM, acetate, and lactate on microtubule protein expression in Caco-2 cells. Protein expression of α-tubulin treated with (**A**) fermented PM, (**B**) acetate, and (**C**) lactate in Caco-2 cells. The cells were treated with fermented PM (0, 1, 2.5, and 5%), acetate (0, 5, 10, 20, and 30 mM), and lactate (0, 5, 10, 20, 30, and 40 mM) for 48 h (*n* = 3 wells per groups). GAPDH was used as the housekeeping protein. Images are representative of three independent experiments. Results are expressed as mean ± standard error (*n* = 3). * (*p* < 0.05) and ** (*p* < 0.01) indicate significant differences compared with the control (0% and 0 mM).

The treatment of the Caco-2 cells with fermented PM significantly decreased the α-tubulin protein levels compared to the control in a concentration-dependent manner (*p* < 0.01; Figure 6A). Acetate also reduced the α-tubulin protein level at a concentration of 30 mM (*p* < 0.05; Figure 6B). The lactate treatment did not induce the down-regulation of α-tubulin (Figure 6C). Therefore, the fermented PM and a high concentration of acetate seem to be associated with microtubule degradation.

### 3.7. Determination of Apoptosis through Activated Caspase-3 and Disruption of α-tubulin in Caco-2 Cells Treated with Fermented Pistachio Milk and Acetate

**Figure 7:**
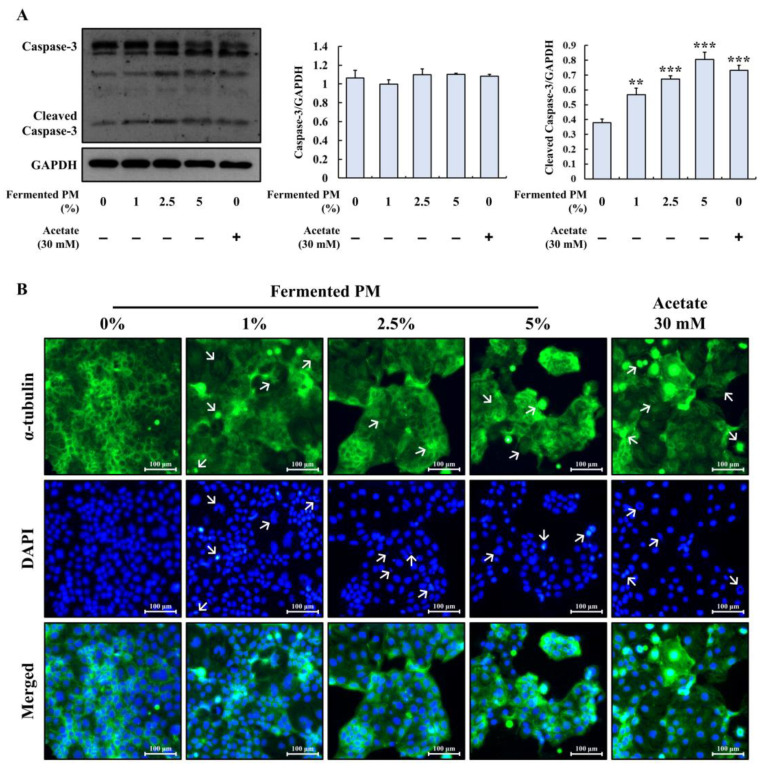
Effects of fermented PM and acetate on apoptosis markers, α-tubulin disruption, and cell nuclei damage in Caco-2 cells. (**A**) Protein expression of caspase-3 and cleaved caspase-3 and (**B**) fluorescence images of α-tubulin and cell nuclei in Caco-2 cells. The magnification of the cell morphology image was 200 ×. Scale bar = 100 µm. White arrows indicate microtubule disruption and aggregation, and the nuclei condensation and fragmentation in Caco-2 cells. The cells were treated with fermented PM (0, 1, 2.5, and 5%) and acetate (30 mM) for 48 h (*n* = 3 wells per groups). GAPDH was used as the loading control. Images are representative of three independent experiments. Results are expressed as mean ± standard error (*n* = 3). ** (*p* < 0.01) and *** (*p* < 0.001) indicate significant differences compared with the control.

To determine the occurrence of apoptosis through caspase-3 activation and microtubule disruption in the Caco-2 cells, the fermented PM (1, 2.5, and 5%) and acetate (30 mM) were used. In the cells treated with the fermented PM, the level of cleaved caspase-3 expression was increased in a concentration-dependent manner (Figure 7A). Acetate (30 mM) also increased the level of cleaved caspase-3 expression compared to that of the control. The pro-caspase-3 expression was not changed by the fermented PM and acetate. Therefore, it seems that the fermented PM and a high concentration of acetate could activate caspase-3 for apoptotic cell death to occur. In the immunofluorescence staining, the fermented PM and acetate induced the disruption and aggregation of α-tubulin as well as nuclear condensation and fragmentation in the Caco-2 cells (Figure 7B). Collectively, the fermented PM and a high concentration of acetate could induce caspase-3 cleavage and microtubule disruption as well as nuclear damage in the Caco-2 cells.

## 4. Discussion

Nuts are rich sources of unsaturated fatty acids, fiber, protein, vitamins, and phytochemical constituents [49]. As a substitute for bovine milk, plant-based milks using nuts such as almonds, cashew nuts, walnuts, and hazelnuts are consumed. Pistachios are abundant in unsaturated fatty acids, fiber, protein, and polyphenols [15]. In addition, pistachios contain lower fat and higher potassium, phytosterol, and vitamins levels in comparison to those of almonds and walnuts. Thus, we used pistachios for plant-based milk production. Plant-based milks are produced through several processes such as separation, homogenization, and thermal processing [50]. These processes cause the loss of many nutrients, especially the dietary fiber. Our data showed that the PM had lower dry matter, protein, fat, ash, and carbohydrate contents than the pistachios did. The protein content of the PM (2.50%) was slightly lower than that in the bovine milk (3.14%), whereas the PM contains a higher protein content than other plant-based milks, including soy milk (2.88%), coconut milk (1.28%), almond milk (0.76%), cashew milk (1.31%), and rice milk (0%) [51,52]. The fat content of the PM was 2.32% higher than that of the bovine milk. In fact, bovine milk contains more saturated fatty acids than it does unsaturated fatty acids [53]. However, the fat in the PM consists of mainly monounsaturated and polyunsaturated fatty acids which are beneficial to cardiovascular health [15,54,55]. The PM had 4.25% lower sugar levels than the bovine milk did since the carbohydrates (sugars and dietary fiber) in pistachios were almost completely removed during the extraction of PM from the pistachios. In our study, to supplement the low sugar content in the PM, inulin (4%) was added to the PM during the fermentation process. Inulin is widely used as an additive for plant-based milk when one supplements the sugar content and fortifies the prebiotics [56,57].

The pH of the fermented PM groups (i.e., C-La, C-Lg, and C-Bb) was slightly higher during fermentation than that of the control group (i.e., C). As in our data, it was reported that co-culture of *S. thermophilus* and *L. bulgaricus* could complete the process of fermentation in the presence of inulin quicker than the other combinations of probiotics could [58,59,60]. In fact, the fermented PM groups (C-La, C-Lg, and C-Bb) had a slightly lower V_max_ value and higher t_max_, t_ph5.0_, and t_f_ values than the control group (C) did. With the same combinations of probiotics, the fermented PM showed a faster fermentation rate and a shorter fermentation completion time than those of fermented bovine milk [24]. This is probably because pistachios possess phenolic compounds that are absent in bovine milk [61]. Phenolic compounds are known to promote the growth of probiotics [62]. 

Our data showed that the fermented PM with inulin contains higher acetate, butyrate, and lactate levels than the fermented PM without inulin does. Heterofermentative lactic acid bacteria such as *Lactobacillus* strains and *Bifidobacterium* strains can metabolize hexoses into acetate and lactate via the phosphoketolase pathways, including the 6p-gluconate pathway and bifidus pathway [63]. Furthermore, *Lactobacillus* ssp. and *Bifidobacterium* ssp. are known to produce acetate and lactate mainly through the degradation of non-digestible carbohydrates, such as inulin [64,65,66]. Meanwhile, the fermented PM without inulin has a similar concentration of acetate and a higher concentration of lactate when it is compared to dairy yogurt with the same combinations of probiotics [24]. The fermented PM had a higher level of butyrate in the presence of inulin, while its propionate content was lower. It was reported that propionate could be maintained or degraded when the inulin is fermented with colonic bacteria [67,68]. Additionally, propionate can be degraded into acetate by the colonic bacteria [69]. In colon microbiota compositions, the intake of inulin could increase the number of *Lactobacillus* ssp. and *Bifidobacterium* ssp. [70,71]. Therefore, the decrease in the amount of propionate in the fermented PM may be due to the degradation by the action of used probiotics belonging to *Lactobacillus* ssp. and *Bifidobacterium* ssp. (i.e., *L. bulgaricus*, *L. acidophilus*, *L. gasseri*, and *B. bifidum*). In the presence of inulin, the fermented PM showed significantly higher concentrations of acetate (3.70~5.58 mmol/L) and lactate (5.15~5.58 mmol/L). These concentrations were markedly higher than the concentrations of butyrate (0.22~0.42 mmol/L). Lactate production was not influenced by the combination of probiotics. However, the amount of acetate production was higher in the combination of *S. thermophilus*, *L. bulgaricus*, and *B. bifidum* (C-Bb). While there is no perfect explanation regarding this acetate production by the combination of probiotics, one possible explanation for it is that *Lactobacillus* strains can degrade inulin-type fructans into short-chain length fractions of inulin, and this can be metabolized into acetate by *Bifidobacterium* strains [64].

The acetate-rich fermented PM decreased the viability of the Caco-2 cells in a concentration-dependent manner. Since the fermented PM is a mixture of fermentation products, it is necessary to identify the substances involved in cell death. The fermented PM is rich in acetate, and thus, acetate alone was also used as a control in the cell viability assay. Our data show that acetate alone also decreased the viability of the Caco-2 cells in a concentration-dependent manner. These data indicate that acetate in the fermented PM plays a role in the cell death of carcinoma cells. In addition, since the fermented PM is also rich in lactate, the cytotoxic effects of lactate itself were observed. The results indicate that death of the cells treated with lactate occurred in a concentration-dependent manner. Collectively, both acetate and lactate may be strongly associated with the observed cytotoxicity on the Caco-2 cells. We confirmed the cytotoxicity using microscopic observations. The Caco-2 cells treated with the fermented PM, acetate, and lactate showed morphological changes (i.e., rounded and shrunk shape) and cellular damage (i.e., detached and floated cells). The rounded and shrunk morphologies of the Caco-2 cells are typically apoptotic characteristics. These apoptotic morphologies can be induced by the condensation of the cell nucleus [72]. For the identification of apoptosis in the Caco-2 cells, we conducted an additional flow cytometry apoptotic cell death test. In the flow cytometry test, the fermented PM increased the number of early and late apoptotic cells in a concentration-dependent manner. Both acetate and lactate also induced early and late apoptosis in the cells. Meanwhile, the apoptosis ratio of the fermented PM was higher than that of acetate and lactate controls. It seems that other factors might be involved in the observed apoptosis in the Caco-2 cells. Pistachios contain high levels of polyphenolic compounds, especially syringic acid and gallic acid [73]. Phenolic acids including syringic acid and gallic acid induced caspase-3 activity in the Caco-2 cells [74]. The fermented PM contains phenolic acids along with acetate and lactate. Collectively, it is assumed that the phenolic acids are also involved in apoptotic cell death in the Caco-2 cells. 

SCFAs and lactate have been known to induce the inhibition of HDAC, and in turn, apoptosis in cancer cells [33,75]. However, acetate and lactate have a weak HDAC inhibition potency. Moreover, SCFAs can influence the formation of microtubules, and this change can induce apoptosis in colon carcinoma cells [36,37,38,39]. Thus, we tested the α-tubulin protein expression in the Caco-2 cells treated with the fermented PM, acetate, and lactate. In the cells treated with the fermented PM and acetate, we found that the α-tubulin protein expression was significantly decreased. Lactate did not change the protein expression of α-tubulin. Thus, it seems α-tubulin is a mediator of apoptosis in the Caco-2 cells, particularly when acetate is the apoptosis-inducing substance. In the same context, acetate seems to be a major apoptosis-inducing substance in fermented PM. α-tubulin is a component of microtubules, along with β-tubulin. As a key component of the cytoskeleton, the microtubules involve mitotic spindle formation, organelles positioning, and cellular motility [34]. The disruption of the microtubules can occur due to α-tubulin degradation, and this consequently causes apoptotic cell death in cancer cells [37,76]. In fact, the mechanisms of apoptosis through down-regulating the α-tubulin protein expression by acetate are not well studied in cancer cells. We suggest new insight into the apoptosis mechanism on Caco-2 cells by acetate and fermented PM that is rich in acetate.

Caspase-3 has been known as an essential player in apoptosis. Activated caspase-3 induces apoptosis via the degradation of the main cellular proteins, DNA fragmentation, and chromatin condensation [77]. Moreover, previous studies have reported that activated caspase-3 could cause the cleavage of α-tubulin, leading to apoptosis [76,78,79]. Our data also demonstrated that the fermented PM and acetate groups induced the up-regulation of cleaved (i.e., active) caspase-3 expression in the Caco-2 cells. The fermented PM also down-regulated the α-tubulin expression. Thus, it seems that the acetate-rich fermented PM could induce the activation of caspase-3, and in turn, caspase-3-mediated α-tubulin degradation. In our data, the α-tubulin disruption was visualized through immunofluorescence staining. The Caco-2 cells treated with the fermented PM revealed a concentration-dependent disruption of α-tubulin. In the acetate (30 mM) treatments, the cells also showed α-tubulin disruption. The level of α-tubulin decrease in the cancer cells could lead to microtubule disruption due to the inhibition of heterodimer (i.e., α- and β-tubulin) formation [39,80]. The microtubule disruption could thus contribute to cell apoptosis. In addition, the fermented PM and acetate groups induced the aggregation of α-tubulin around the cell nuclei, and consequently, cytoplasmic retraction and their rounded shape. Previous studies have also shown that α-tubulin disruption and aggregation were observed in cancer cells that had undergone caspase-3-mediated apoptosis [76,78]. Along with α-tubulin disruption, our data also demonstrate that the fermented PM and acetate groups induced nuclear condensation and fragmentation. These nuclear damages are typical features of apoptosis. Therefore, the acetate-rich fermented PM could induce apoptosis in the Caco-2 cells through capsase-3 activation, microtubule disruption, and nuclear damage.

## 5. Conclusions

Pistachio milk, a non-dairy beverage derived from pistachios, seems to contain abundant levels of protein and fat, and these are useful in the development of a fermented product due to its short fermentation time. The fermentation of pistachio milk supplemented with 4% inulin using *S. thermophilus*, *L. bulgaricus*, and *B. bifidum* could produce a high amount of acetate. The acetate-rich fermented pistachio milk exhibits cytotoxicity and apoptotic cell death against colon carcinoma cells, Caco-2, through the microtubule disruption and nuclear damage mediated by caspase-3. Although additional studies are required, our data suggest that fermented pistachio milk might be a novel therapeutic food in the treatment of colon cancer. Additionally, the consumption of fermented pistachio milk might be beneficial to patients with colon cancer or people at high risk of developing colon cancer.

## Figures and Tables

**Table 1:** The conditions for dry matter and ash content measurements in pistachio, PM, and bovine milk.

Samples	Dry Matter		Ash	
	Initial Weight	Temperature and Time	Initial Weight	Temperature and Time
Pistachio	1 g	105 °C, 24 h	1 g	550 °C, 6 h
PM	2 g	101 °C, 1 h	2 g	550 °C, 1 h
Bovine milk	2 g	101 °C, 1 h	2 g	550 °C, 1 h

**Table 2:** Proximate composition of pistachio, PM, and bovine milk.

Samples	Dry Matter (%)	Protein (%)	Fat (%)	Ash (%)	Total Carbohydrate	
					Sugars (%)	Dietary Fiber (%)
Pistachio	96.22 ± 0.04 ^a^	25.33 ± 0.34 ^a^	43.57 ± 0.19 ^a^	2.60 ± 0.06 ^a^	4.64 ± 0.71 ^a^	20.08 ± 0.80
PM	9.64 ± 0.02 ^c^	2.50 ± 0.06 ^b^	6.37 ± 0.18 ^b^	0.51 ± 0.03 ^c^	0.25 ± 0.09 ^b^	0.00 ± 0.00
Bovine milk	13.37 ± 0.04 ^b^	3.14 ± 0.00 ^b^	4.05 ± 0.00 ^c^	1.68 ± 0.04 ^b^	4.50 ± 0.01 ^a^	- ^1^

^1^ Indicates no data; ^a–c^ Means in each column with different superscripts represent significant difference (*p* < 0.05). Results are expressed as mean ± standard error (*n* = 3).

**Table 3:** Acidification kinetic parameters of fermented PM groups.

Acidification Kinetics Parameters ^1^	Fermented PM Groups ^2^			
	C	C-La	C-Lg	C-Bb
V_max_ (10^−3^ pH units/min)	9.93 ± 0.04 ^a^	9.14 ± 0.05 ^b^	8.91 ± 0.01 ^b^	9.27 ± 0.14 ^b^
t_max_ (h)	1.29 ± 0.01 ^c^	1.55 ± 0.03 ^b^	1.86 ± 0.03 ^a^	1.60 ± 0.01 ^b^
t_pH 5.0_ (h)	2.13 ± 0.03 ^c^	2.28 ± 0.00 ^b^	2.48 ± 0.00 ^a^	2.33 ± 0.01 ^b^
t_f_ (h)	3.34 ± 0.01 ^b^	3.43 ± 0.02 ^ab^	3.50 ± 0.00 ^a^	3.47 ± 0.03 ^a^

^1^ V_max_ = maximum acidification rate; t_max_ = time required to reach V_max_; t_pH 5.0_ = time taken to reach pH 5.0; t_f_ = time taken to complete the fermentation. ^2^ C = PM fermented with *Streptococcus thermophilus* and *Lactobacillus bulgaricus*; C-La = PM fermented with *S. thermophilus*, *L. bulgaricus*, and *Lactobacillus acidophilus*; C-Lg = PM fermented with *S. thermophilus*, *L. bulgaricus*, and *Lactobacillus gasseri*; C-Bb = PM fermented with *S. thermophilus*, *L. bulgaricus*, and *Bifidobacterium*. ^a–b^ Means with different superscripts indicate significant differences among fermented PM groups (*p* < 0.05). Results are expressed as mean ± standard error (*n* = 3).

## Data Availability

Data are contained within the article or Supplementary Materials.

## Supplementary Materials

The following supporting information can be downloaded at: https://www.mdpi.com/article/10.3390/foods12010189/s1, Figure S1: GC chromatogram of acetate (C2), propionate (C3), butyrate (C4), and lactate (L) in PMs and fermented PMs with or without 4% inulin.
