# Supplementary figures and images for: Apoptosis-Inducing Effects of Short-Chain Fatty Acids-Rich Fermented Pistachio Milk in Human Colon Carcinoma Cells
Source: Foods. 2023 Jan 1;12(1):189. doi: 10.3390/foods12010189 (PMC9818824; doi:10.3390/foods12010189)

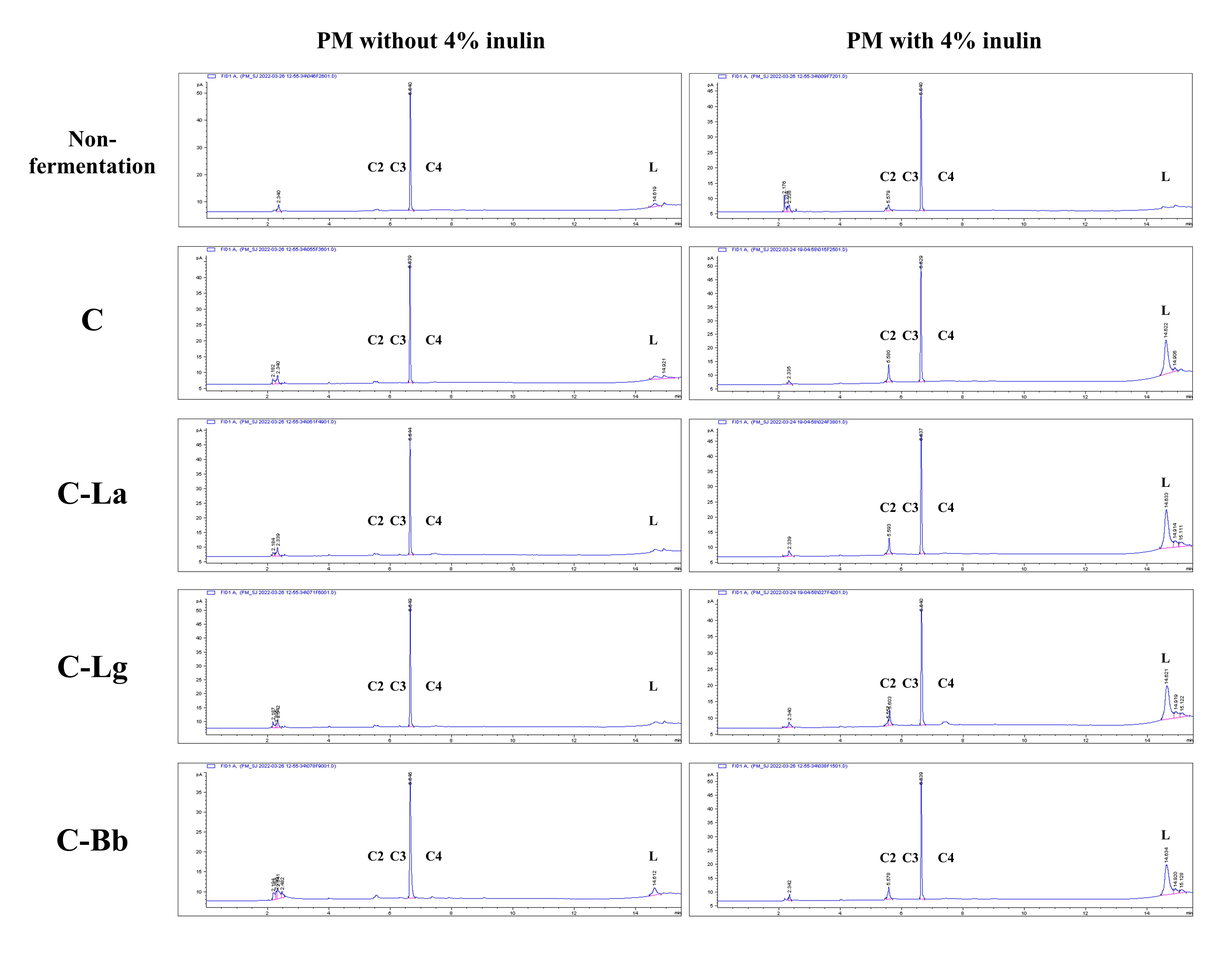

Supplement: Supplementary file 1 [file foods-12-00189-s001.zip › foods-2035981-supplementary.tif]
